# Determination of the Spatial Distribution of Air Pollutants in Bucheon, Republic of Korea, in Winter Using a GIS-Based Mobile Laboratory

**DOI:** 10.3390/toxics11110932

**Published:** 2023-11-16

**Authors:** Minkyeong Kim, Daeho Kim, Yelim Jang, Jooyeon Lee, Sangwon Ko, Kyunghoon Kim, Choonsoo Park, Duckshin Park

**Affiliations:** 1 Railroad Test & Certification Division, Korea Railroad Research Institute (KRRI), Cheoldo Bangmulgwanro, Uiwang-si 16105, Republic of Korea; mkkim15@krri.re.kr; 2 Department of Environmental Science and Ecological Engineering, Korea University, Seoul 02841, Republic of Korea; daehoya@gmail.com; 3 Chemicals Research Division, Environmental Health Research Department, National Institute of Environmental Research, Incheon 22689, Republic of Korea; yelimm412@korea.kr; 4 Transportation Environmental Research Department, Korea Railroad Research Institute (KRRI), Cheoldo Bangmulgwan-ro, Uiwang-si 16105, Republic of Korea; jooyeon07@krri.re.kr (J.L.); sko@krri.re.kr (S.K.); khkim20@krri.re.kr (K.K.); 5 New Transportation Innovation Research Center, Korea Railroad Research Institute (KRRI), Cheoldo Bangmulgwan-ro, Uiwang-si 16105, Republic of Korea; cspark@krri.re.kr

**Keywords:** air pollution, geographic information system, hotspot analysis, mobile laboratory, spatial distribution characteristics

## Abstract

Driven by industrialization and urbanization, urban air pollution can increase respiratory, heart, and cerebrovascular diseases, and thus mortality rates; as such, it is necessary to improve air quality through the consideration of individual pollutants and emission sources. In Republic of Korea, national and local governments have installed urban and roadside air quality monitoring systems. However, stations are lacking outside metropolitan regions, and roadside stations are sparsely distributed, limiting comparisons of pollutant concentrations with vehicle traffic and floating population levels. Local governments have begun using mobile laboratories (MLs) to supplement the fixed measurement network and investigate road pollution source characteristics based on their spatiotemporal distribution; however, the collected data cannot be used effectively if they are not visualized. Here, we propose a method to collect and visualize global information system (GIS)-based air quality data overlayed with environmental variables to support air quality management measures. Spatiotemporal analyses of ML-derived data from Bucheon, Korea, confirmed that particulate and gaseous pollutant concentrations were high during typical commuting hours, at intersections, and at a specially managed road. During commuting hours, the maximum PM_10_ concentration reached 200.7 µg/m^3^ in the Nae-dong, Gyeongin-ro, and Ojeong-dong ready-mix concrete complex areas, and the maximum PM_2.5_ concentration was 161.7 µg/m^3^. The maximum NO_x_, NO_2_, and NO levels of 1.34 ppm, 0.18 ppm, and 1.18 ppm, respectively, were also detected during commuting hours. These findings support the need for targeted management of air pollution in this region, and highlight the benefit of comprehensively comparing road levels, driving speed, and traffic levels when identifying hotspots of air pollution. Such analyses will contribute to the development of air quality management measures customized to regional characteristics.

## 1. Introduction

According to Statistics Korea (2023), more than half of the total Korean population lives in metropolitan areas (e.g., Seoul, Incheon, and Gyeonggi-do). Industrialization and urbanization lead to various social problems, such as high population densities, traffic congestion, and environmental pollution [1,2,3,4]. In particular, rapid densification frequently results in increased air pollutant emissions and increases in temperature, which in turn reduce air quality. Among air pollutants found in urban areas, major pollutants include nitrogen dioxide (NO_2_), carbon monoxide (CO), fine particulate matter (PM_10_, PM_2.5_), black carbon (BC), volatile organic compounds, and ozone (O_3_), which are generated through secondary reactions emitted from automobiles. These pollutants can cause respiratory, heart, and cerebrovascular diseases, and increase mortality rates [4,5,6].

To enact effective air pollution reduction policies and measures, governments require spatiotemporal information about air pollutant characteristics. Local governments have started to continuously monitor the status of air pollutants (e.g., PM, O_3_, and yellow dust) that threaten public health, and to secure the basic data necessary to establish air quality improvement measures. In Korea, air pollution monitoring networks include the urban air quality monitoring system (AQMS) and roadside air quality monitoring system (RAQMS) [7,8]. The AQMS monitors atmospheric pollutants and environmental pollutants (e.g., SO_2_, NO_2_, CO, O_3_, PM_10_, and PM_2.5_), as well as atmospheric and meteorological factors, in densely populated areas to improve air quality and environmental policies. The RAQMS is installed along high-traffic roadways to assess air quality along roads and manage the generation of pollutants emitted by vehicles. These systems provide air pollution data in real time that are available online, and help support air environment improvement plans by monitoring air pollution levels.

However, these networks have limitations. Both networks employ fixed measurement stations. Moreover, until 2016, most AQMS stations were installed only near metropolitan areas. Meanwhile, RAQMS stations are sparsely dispersed, so the system cannot capture air pollution or traffic volumes along many roads [4,9]. To compensate for these shortcomings, a mobile laboratory (ML) was introduced in 2011 in Korea to investigate the characteristics of roadside air pollution sources and the spatiotemporal distribution of air pollutants in urban areas [4,10,11,12,13]. MLs are deployed in vehicles, and can survey roads not covered by the monitoring networks, analyze pollution levels in areas around industrial complexes, and conduct real-time surveys of redispersed road dust. These data can then be used to recommend location-specific measures to reduce air pollutant levels [4,5,9,14,15,16]. Most local governments in Korea are expanding the use of ML vehicles and drones to monitor air quality in areas without existing monitoring stations. However, in many cases, the collected data are not effectively visualized. To improve analyses of air pollution hotspots, there is a need to advance air pollution management through geographic information system (GIS)-based data visualization, digital twin models, and overlayed environmental factor data. In particular, GIS-based spatial analyses can enhance the interpretation of spatial phenomena and support rational, locally customized decision making [17].

This study is the first to use ML-derived data to visualize the relationships among GIS-based environmental variables to improve air quality assessment and management. We used Bucheon, Korea, as the study area because of its high traffic volume and dense urban population. Using an ML equipped with real-time measurement equipment, we determined the spatiotemporal distributions of major air pollutants along a route that passed through residential and industrial areas. Using GIS-based data visualization to identify pollution hotspots, we offer policy recommendations to improve local air management measures. This data acquisition and analysis method has broad applicability.

## 2. Materials and Methods

### 2.1. Research Site

As of 2020, Bucheon, Korea, has an area of 53.4 km^2^, a population of 831,701, and a population density of 15,575 people/km^2^. It has the highest population density in Gyeonggi-do, and contains a high concentration of residential and industrial facilities. In addition, it is located between Seoul and Incheon, adjacent to the Gyeongin Expressway and the Capital Region First Ring Expressway; the traffic volume is 3.3 million people per day, which is very high compared to its population. There are four AQMS stations (Nae-dong, Sosabon-dong, Ojeong-dong, and Jung 2-dong) and one RAQMS station (Songnae-daero) in Bucheon. These stations measure a total of 10 primary pollutants.

**Figure 1:**
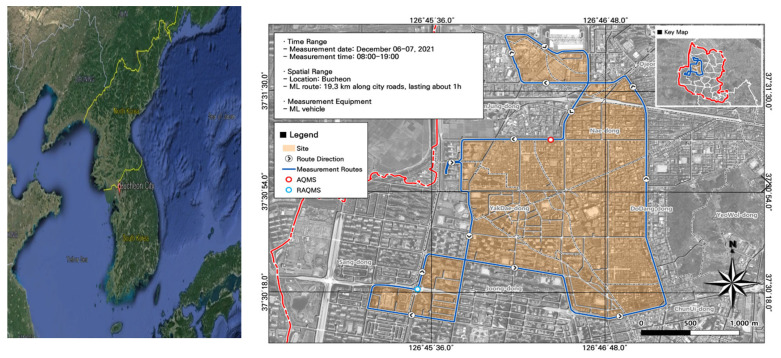
Location of the study site, mobile laboratory (ML) route, and fixed air quality monitoring system (AQMS) and roadside air quality monitoring system (RAQMS) stations.

To select our study site, we reviewed the results of a hotspot analysis from the literature [4]. The site was adjacent to the Gyeongin Expressway and the Capital Region First Ring Expressway, and also near the Nae-dong AQMS station and Songnae-daero RAQMS station. We selected a survey route that passed near industrial complexes and a specially managed road (roads with severe traffic congestion) (Figure 1).

### 2.2. Experimental Methods

#### 2.2.1. Mobile Laboratory

**Figure 2:**
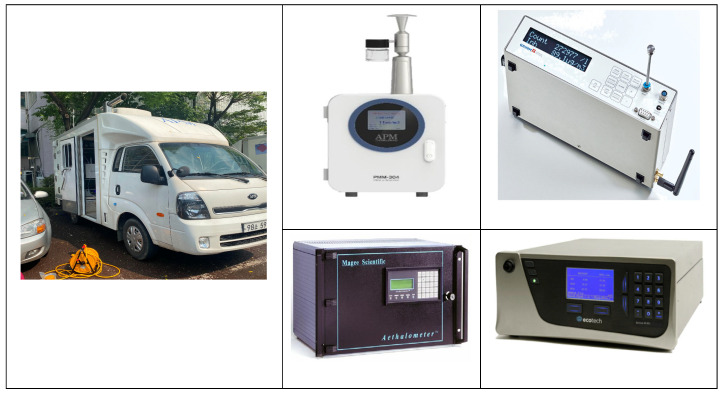
Air pollution mobile laboratory vehicle (**left**) and measurement equipment: APM PMM-304 (**upper middle**), Grimm 11-D (**upper right**), Magee Scientific AE51 aethalometer (**lower middle**), and Ecotech Serinus 40 (**lower right**).

We used an ML to measure location information and air pollutants (Figure 2). MLs enable the real-time measurement of spatiotemporal distributions and concentrations of air pollutants within a target area. However, there are time delays between the flow of pollutants into the inlet and measurement, difficulties in uniform absorption due to the physical characteristics of different pollutants, and separation from GPS data due to equipment sensitivity. Regardless, MLs are widely used, and the limitations can be compensated for according to the engineering design of the inlet [4,18,19,20,21,22].

For gas-phase pollutants, we followed the so-called Korean air pollution process test method. This method recommends using gravimetry and beta-ray absorption to measure fine dust in the atmosphere; however, for mobile measurements, we used light-scattering measurement methods to obtain faster responses. Black carbon (BC) is used as an indicator of automobile emission pollution; we measured BC using an AE51 aethalometer (Magee Scientific, Berkley, CA, USA). We also used a GPS system to determine the spatial coordinates of measurements according to vehicle movement to prevent measurement errors due to time differences among measurement devices (Table 1).

**Figure 3:**
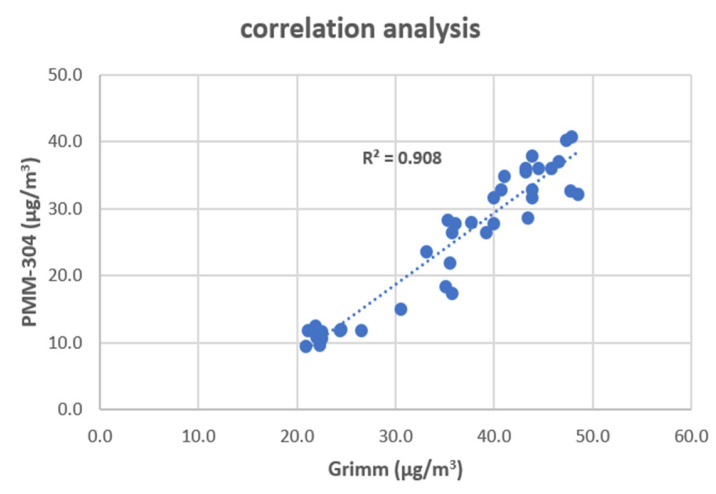
Correlation analysis of the fine particulate matter datasets obtained from the PMM-304 and Grimm 11-D instruments.

PM was measured using a PMM-304 (APM, Bucheon, Republic of Korea) and Grimm 11-D (Grimm Aerosol Technik, Berlin, Germany). The PMM-304 measured PM_2.5_ at 60 s intervals, and the Grimm 11-D measured PM_2.5_ and PM_10_ at 6 s intervals. Comparison of the two datasets revealed a high correlation (R^2^ = 0.908) (Figure 3). Both devices have high accuracy, but for the spatiotemporal analysis, we used data from the Grimm 11-D, which were collected at a shorter interval.

The ML vehicle was remodeled after approval of the structural changes. The inside of the vehicle was equipped with equipment and a generator to measure PM. An intake port was installed on the roof of the vehicle to collect air samples while driving; it was designed to collect particulate pollutants at a constant velocity. At vehicle speeds of 0–70 km/h, we confirmed the average speed to have a rate of change of less than 0.8–2.0%; therefore, measurements in traffic were conducted within this speed range as much as possible [23].

#### 2.2.2. Measurement Procedure

The mobile measurements were taken along a predetermined route, which started and ended at the Bucheon Technopark, Yakdae-dong; before operation, the equipment was inspected and the battery was charged.

Beginning at the Technopark, the route passed through Gyenam Park and the Eugene ready-mixed concrete complex, and near Songnae-daero, Gilju-ro, Gyenam-ro, and the Gyeongin Expressway. The route was 19.3 km, and took about 1 h. Because fine dust concentrations are highest from December to March, the study was conducted on 6 and 7 December. We conducted seven sets of mobile measurements (five on 6 December, and five on 7 December) during regular commuting and work hours. To ensure a constant velocity of PM with the inlet, the driving speed was maintained around 20–30 km/h.

#### 2.2.3. GIS Analysis

We used the open-source software QGIS ver. 3.32 to analyze the spatial and temporal distributions of air pollution in Bucheon. The GIS analysis was based on the GPS-derived coordinates of the ML and the time and concentration data for the measured pollutants.

**Figure 4:**
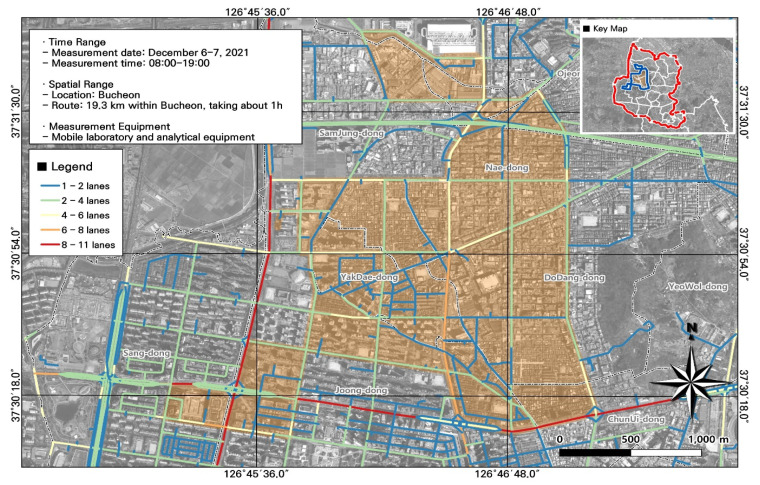
Number of lanes of roads within the study area.

Most of the study area was urbanized, so land-cover variables were limited. Instead, we collected data on road information, vehicle speed (e.g., average speed during congestion and maximum speed), and traffic volume as important variables to assess the particulate and gaseous pollutants generated on roads. We used openly available data in the View-T service from the National Transport Database, Korea Transport Institute (Figure 4).

## 3. Results

### 3.1. Particulate Pollution in Bucheon

We performed a comparative analysis of the ML-derived data from the study area and data from nearby AQMS and RAQMS stations (Table 2). The area included residential and industrial complexes, so the spatial distributions of pollutant concentrations varied markedly.

The ML-measured PM_10_ (78.7 ± 26.2 µg/m^3^) and PM_2.5_ (55.7 ± 13.0 µg/m^3^) concentrations were similar to those of the AQMS, but RAQMS was significantly different for PM_10_ (63.5 ± 12.6 µg/m^3^) and PM_2.5_ (38.9 ± 8.3 µg/m^3^) concentrations, with errors of 15–17 µg/m^3^. Sources of particulate pollutants include not only automobile exhaust but also re-dispersed road particulates, pollen, and long-range transport from other regions. The urban particulate (PM_2.5_, PM_10_) levels from the AQMS were similar to the ML-derived roadside concentrations, indicating that there were no additional influencing factors along the road. Real-time BC measurements along roads have recently been used as an indicator of automobile emissions; the average BC concentration was 6205.7 ± 3020.1 µg/m^3^ as measured using the ML. The AQMS and RAQMS stations do not measure BC, so comparison was not possible.

The ML-derived concentrations of the gaseous pollutants NO, NO_2_, and NO_x_ were 0.21 ± 0.16 ppm, 0.07 ± 0.03 ppm, and 0.28 ± 0.18 ppm, respectively. Automobile exhaust is the main source of NO_x_ emissions in cities; therefore, their emissions are often higher along roads than at urban measurement stations. Accordingly, the ML-measured NO_2_ levels in this study were higher than those from the AQMS and RAQMS.

Urban pollution levels are much higher near high-volume intersections than surrounding areas, and decrease with increasing distance from roads. The study route included a specially managed road, so we could assess whether particulate and gaseous pollutants were correlated with road emission sources.

### 3.2. Spatiotemporal Patterns of PM_2.5_ and PM_10_ Concentrations

**Figure 5:**
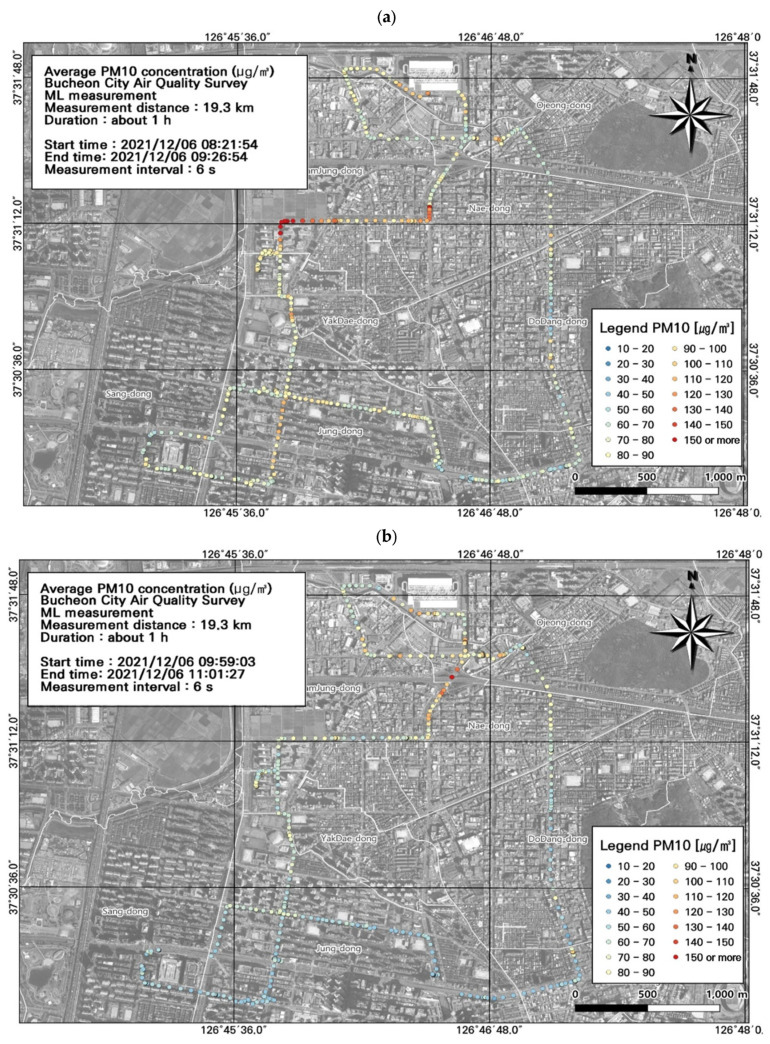

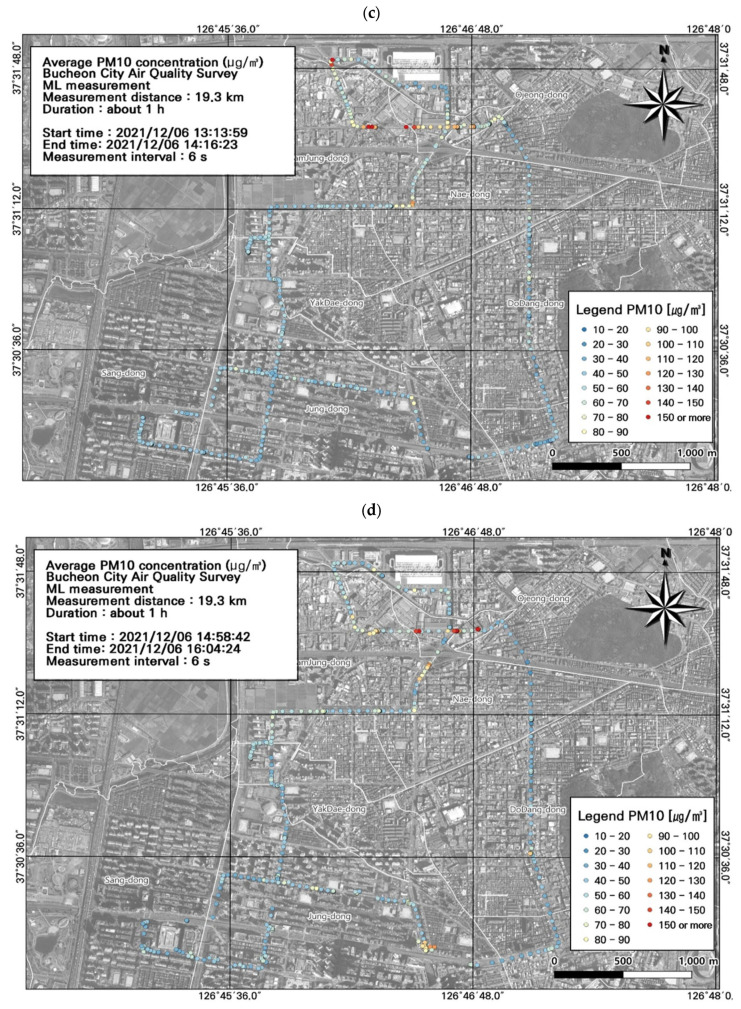

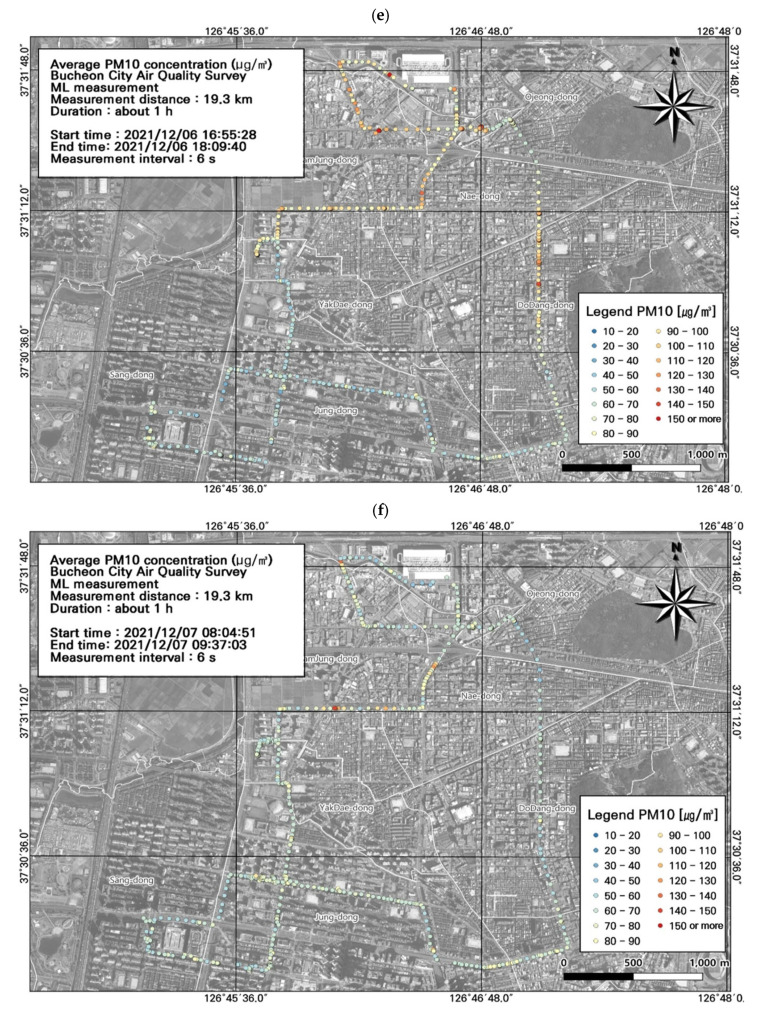

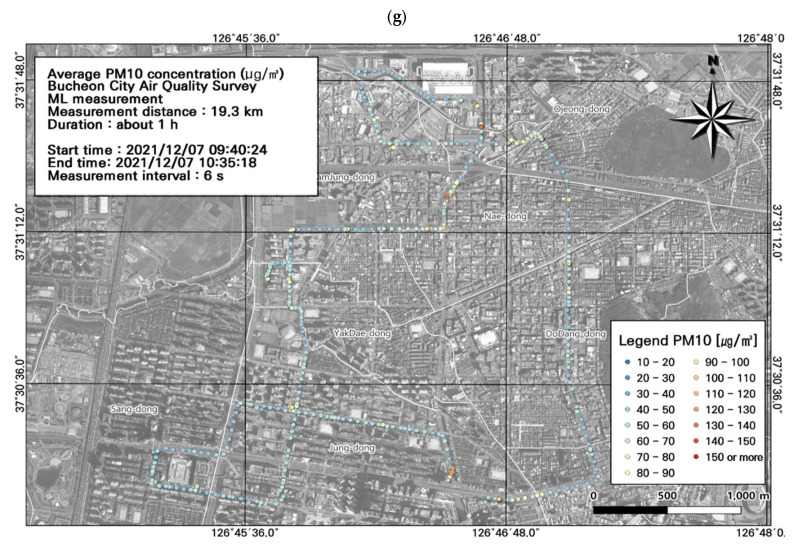
Spatial and temporal plots of particulate matter (PM_10_) concentrations measured using the mobile laboratory (ML): (**a**) 6 December, first run; (**b**) 6 December, second run; (**c**) 6 December, third run; (**d**) 6 December, fourth run; (**e**) 6 December, fifth run; (**f**) 7 December, first run; (**g**) 7 December, second run.

Among the seven measurement runs, the highest PM_10_ concentration (91.8 ± 17.6 µg/m^3^) was detected during typical evening rush hour, followed by morning rush hour (79.6 ± 19.7 µg/m^3^) (Figure 5a,e; Table 3). The spatiotemporal distributions confirmed high PM_10_ concentrations during commuting hours in the areas near the Nae-dong, Gyeongin-ro, and Ojeong-dong ready-mix concrete complex. During morning rush hour, the highest PM_10_ concentration was 190 µg/m^3^ in the Nae-dong and Gyeongin-ro areas. During evening rush hour, the highest PM_10_ concentration was 200.7 µg/m^3^ in the areas of the Nae-dong, Gyeongin-ro, and Ojeong-dong ready-mix concrete complex. These findings confirm high contributions of roadside mobile emissions to air pollution; accordingly, careful management of these areas is necessary during commuting hours.

The 1 h average measurements from the AQMS can be checked in real time, but the measurement interval limits their comparison with nearby traffic variables. To overcome this limitation, we used the ML-derived pollutant concentrations (collected in the order of seconds) to compare the spatiotemporal distributions with traffic variables.

**Figure 6:**
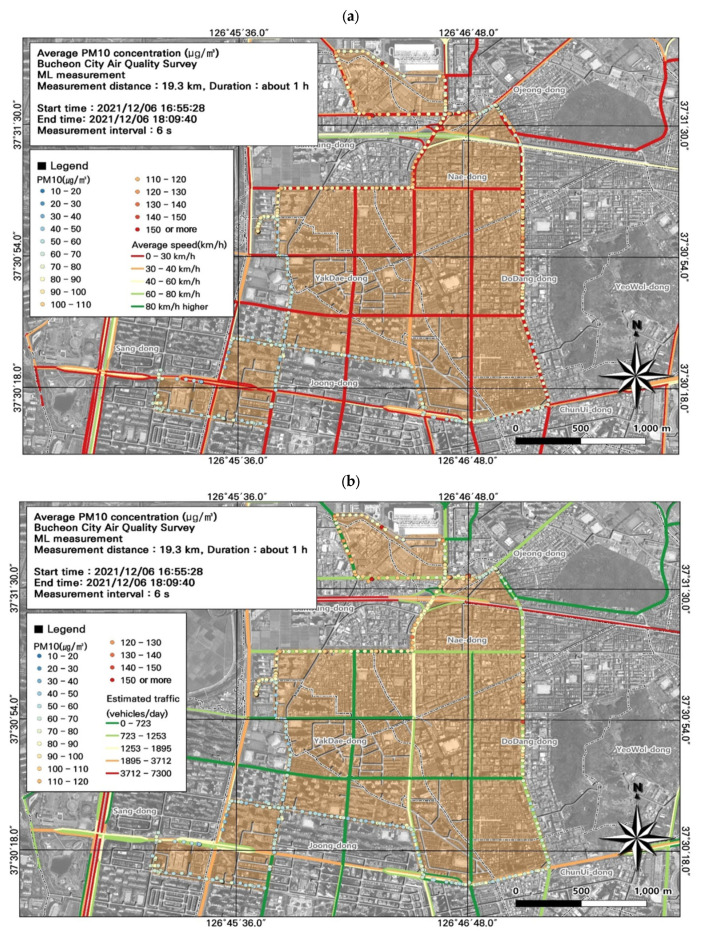
Association between particulate matter (PM_10_) concentrations with (**a**) average traffic speed and (**b**) estimated traffic volume during the evening rush hour from Figure 5e.

Overall, the study area had high PM_10_ concentrations, and we performed a spatial analysis of the data collected during work hours (Figure 5), when concentrations were high along the entire route (Figure 6). Based on the ML vehicle speed, we found that most of the route experienced traffic congestion during commuting hours, during which time the average speed was 0–30 km/h. The PM_10_ concentration was highest during morning rush hour in the Nae-dong and Gyeongin-ro areas (190 µg/m^3^), and during evening rush hour in the Nae-dong, Gyeongin-ro, and Ojeong-dong ready-mix concrete complex areas (200.7 µg/m^3^). Comparison of the estimated traffic volume and PM_10_ concentration values confirmed that the traffic volume was elevated (723–1253 vehicles/day) near Nae-dong and Gyeongin-ro, where PM_10_ concentrations were relatively high. These findings indicate that road emissions influence PM_10_ concentrations.

**Figure 7:**
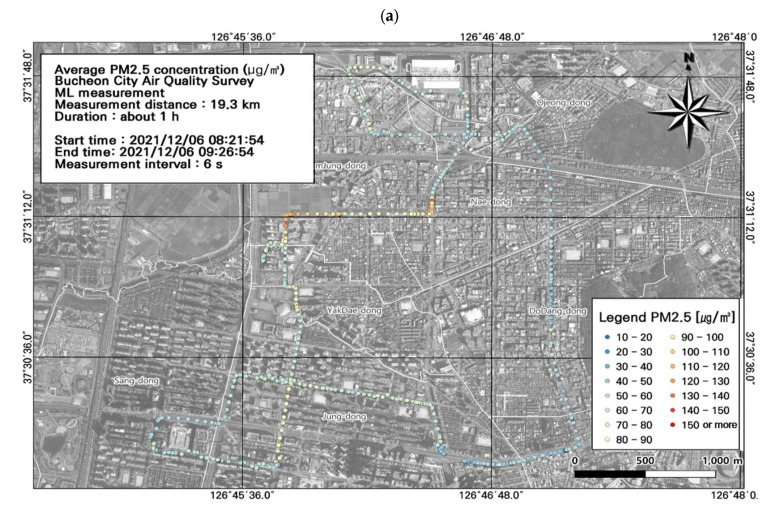

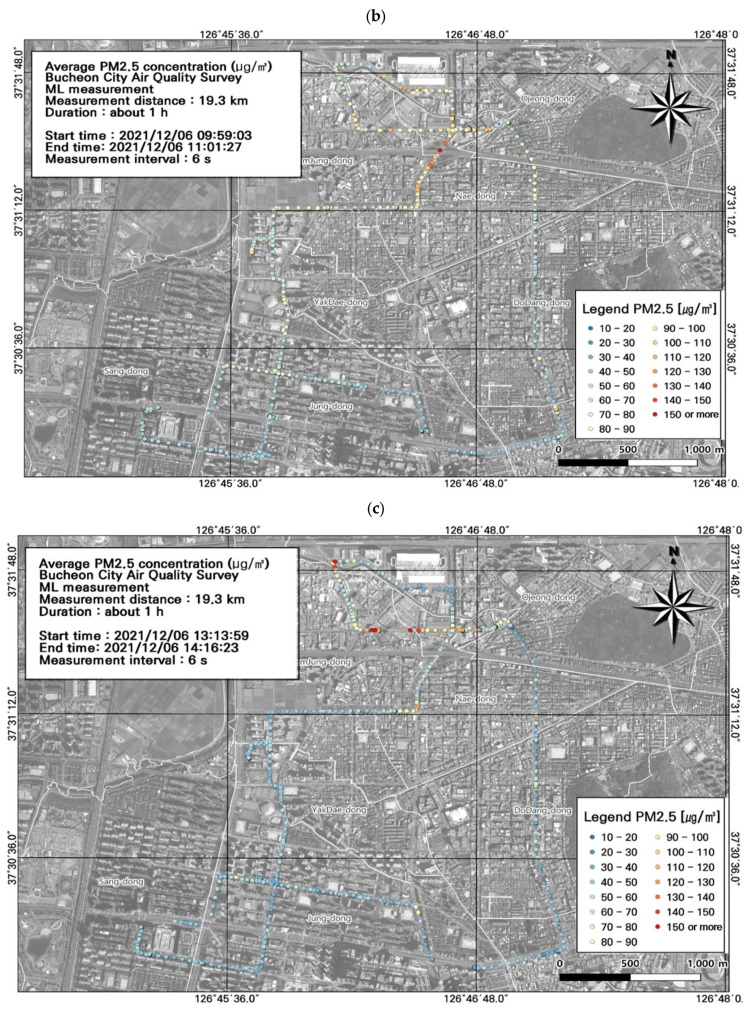

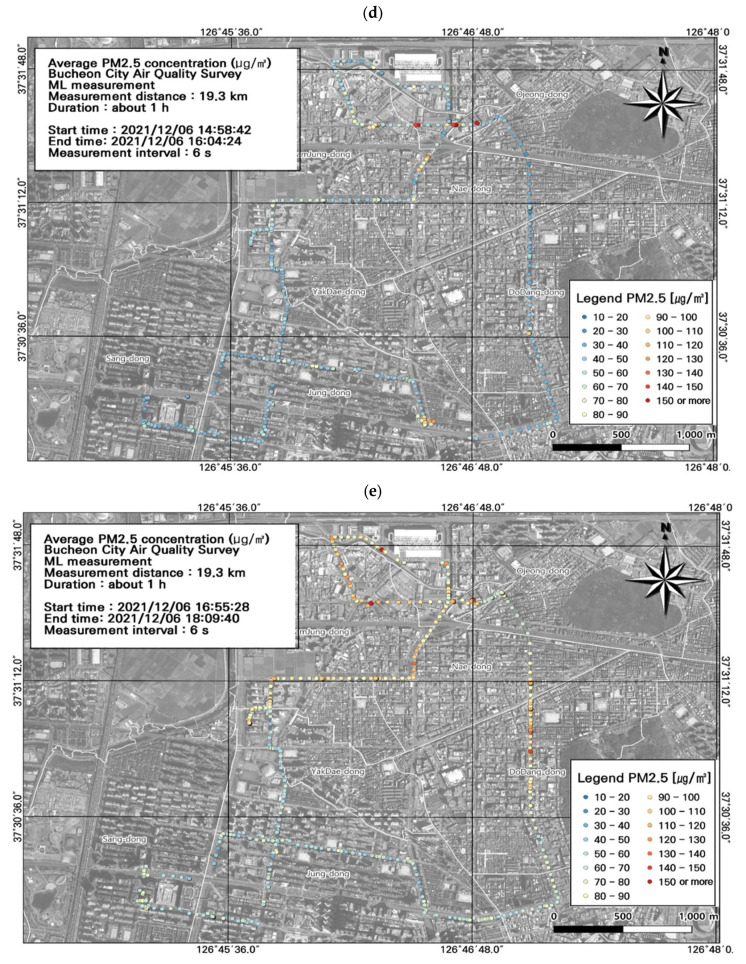

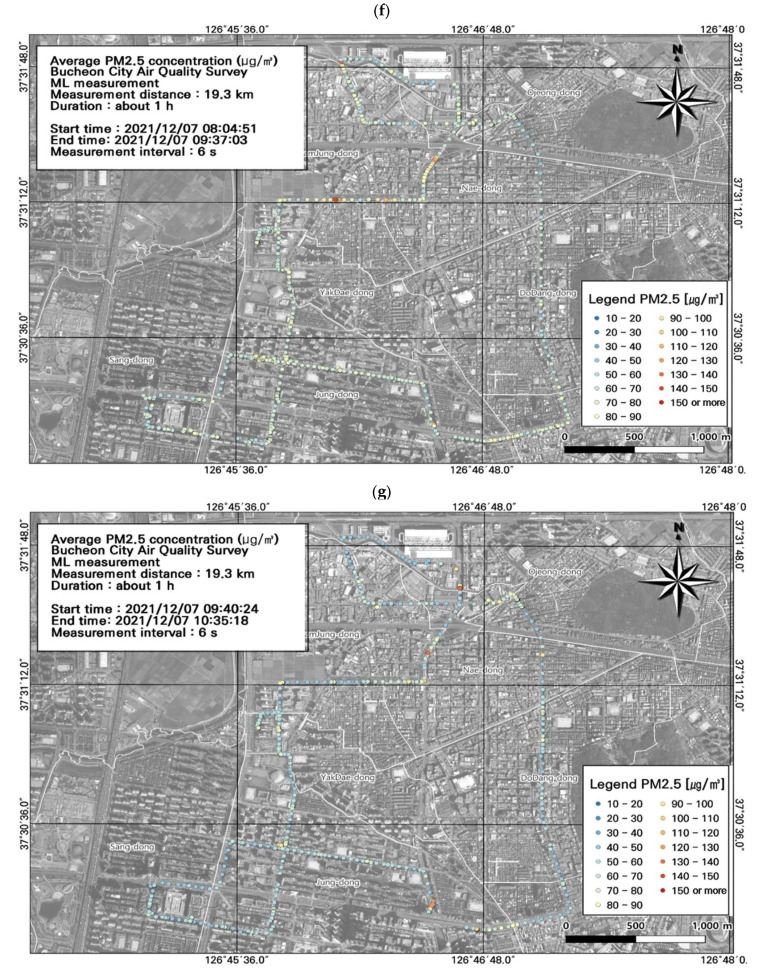
Spatial and temporal plots of ultrafine particulate matter (PM_2.5_) concentrations measured using the mobile laboratory (ML): (**a**) 6 December, first run; (**b**) 6 December, second run; (**c**) 6 December, third run; (**d**) 6 December, fourth run; (**e**) 6 December, fifth run; (**f**) 7 December, first run; (**g**) 7 December, second run.

Among the seven ML datasets of PM_2.5_ concentrations, the highest average concentrations were measured during the two morning rush hours (day 1: 62.7 ± 3.0 µg/m^3^; day 2: 59.5 ± 6.8 µg/m^3^) (Figure 7), followed by the evening rush hour (59.2 ± 8.8 µg/m^3^) (Figure 7). During commuting hours, the highest PM_2.5_ concentration was 132.9 µg/m^3^. During work hours, the highest PM_2.5_ concentration was 161.7 µg/m^3^ in the areas of the Nae-dong, Gyeongin-ro, and Ojeong-dong ready-mix concrete complex. Similar to the PM_10_ results, these findings confirmed high PM_2.5_ concentrations during commuting hours, and thus high contributions from mobile emissions, particularly near the Nae-dong, Gyeongin-ro, and Ojeong-dong ready-mix concrete complex. Accordingly, careful management of these areas is necessary during commuting hours.

**Figure 8:**
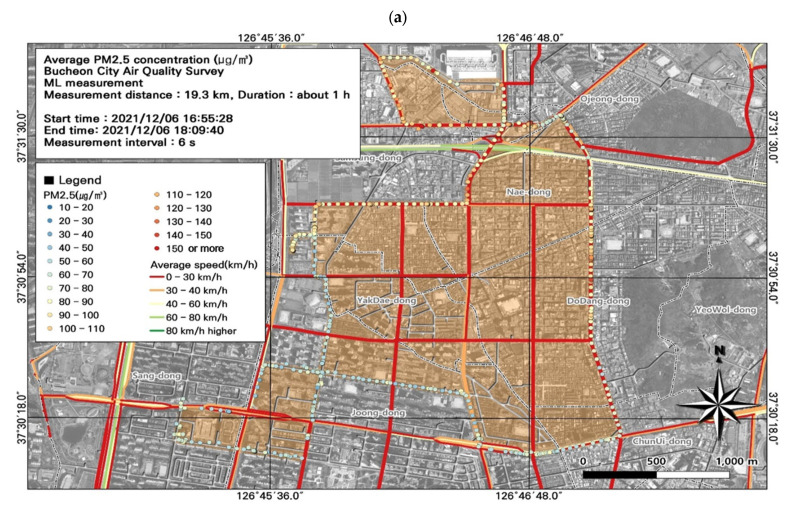

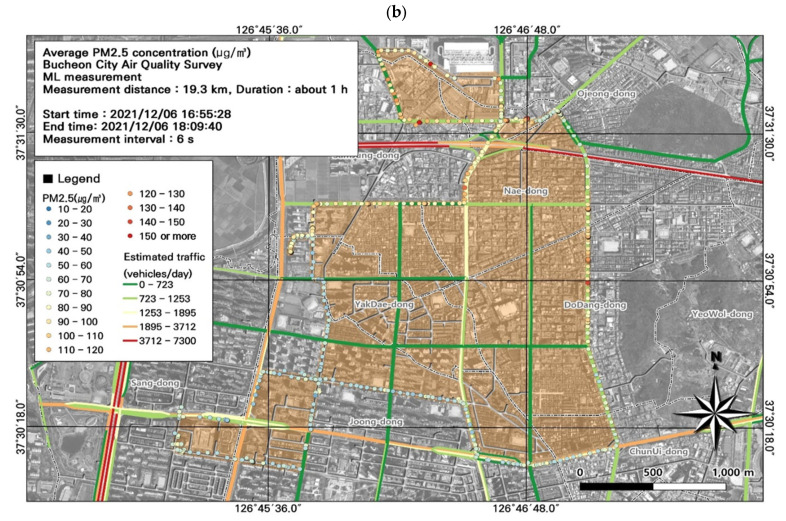
Association between fine particulate matter (PM_2.5_) concentrations and (**a**) average traffic speed and (**b**) estimated traffic volume during the evening rush hour from Figure 7e.

Overall, the study area had high PM_2.5_ concentrations, and we performed a spatial analysis of the data collected during work hours (Figure 7), when concentrations were relatively high along the entire route (Figure 8). As noted for the PM_10_ results, most of the route was congested during commuting hours, and the ML vehicle average speed was 0–30 km/h. The highest PM_2.5_ concentration during commuting hours (132.9 µg/m^3^) was measured in the Nae-dong and Gyeongin-ro areas, and during work hours (161.7 µg/m^3^) was measured in the areas of the Nae-dong, Gyeongin-ro, and Ojeong-dong ready-mix concrete complex. Comparison of the PM_2.5_ concentrations with estimated traffic volume revealed high PM_2.5_ concentrations near areas with elevated traffic volumes (723–1253 vehicles/day), i.e., Nae-dong and Gyeongin-ro. The traffic volume was also relatively high near the Ojeong-dong ready-mix concrete complex, which experienced high PM_2.5_ concentrations. Thus, road emissions impact both PM_10_ and PM_2.5_ concentrations.

To examine the impact of automobiles on the spatiotemporal distributions of PM_10_ and PM_2.5_ concentrations, it is necessary to comprehensively review road and traffic volume variables using GIS. Such analyses allow hotspots to be identified for targeted air quality management measures.

### 3.3. Spatiotemporal Patterns of BC Concentrations

**Figure 9:**
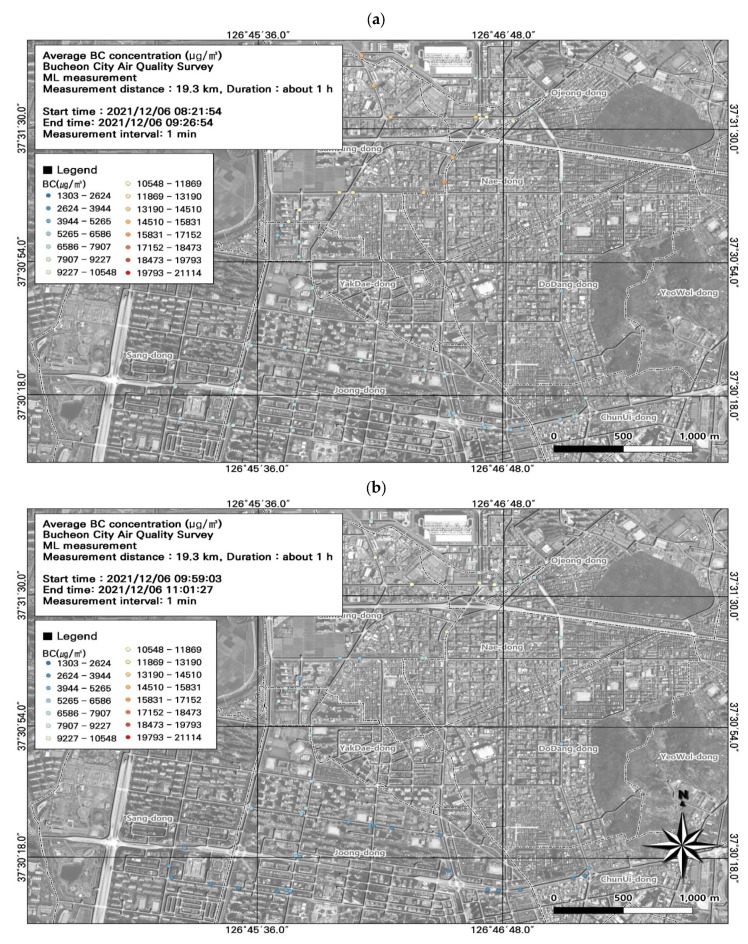

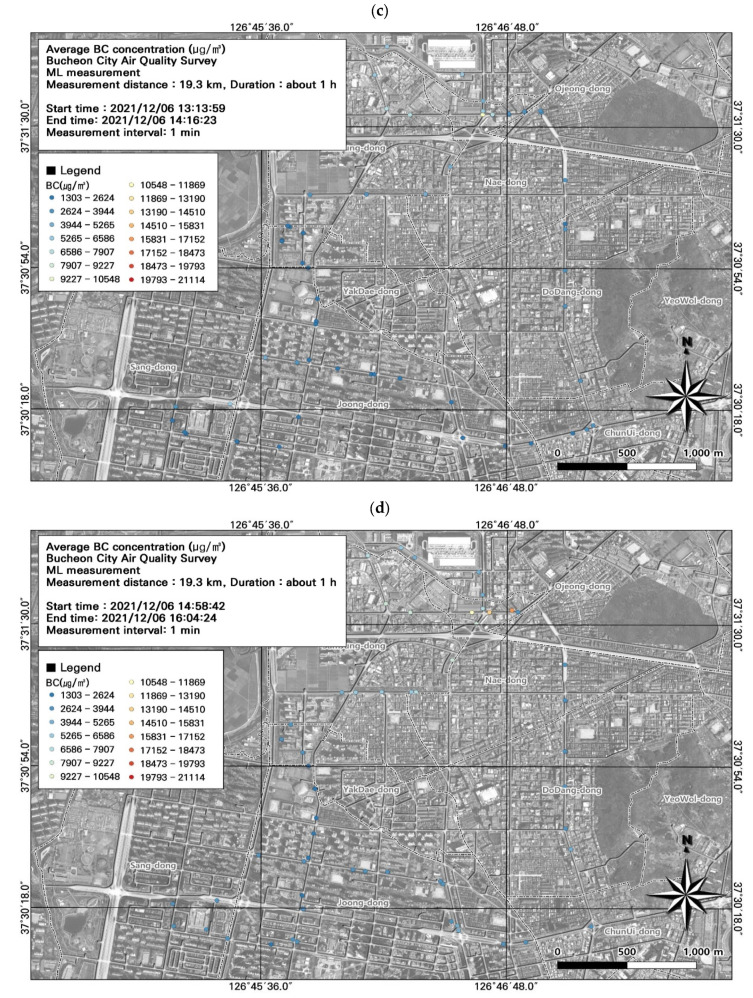

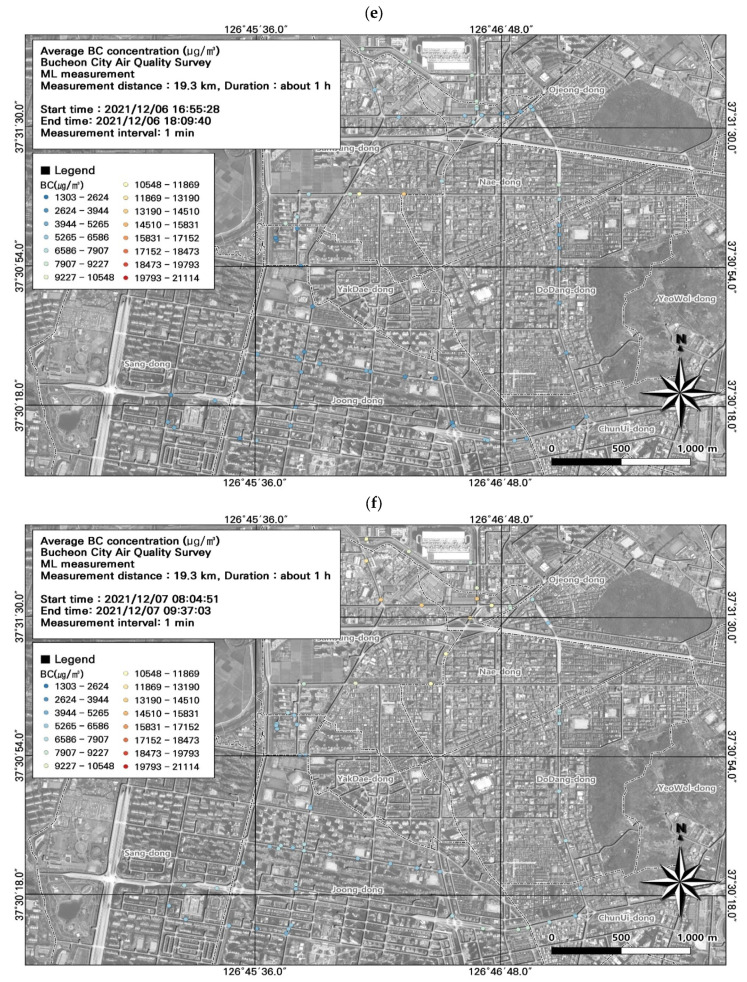

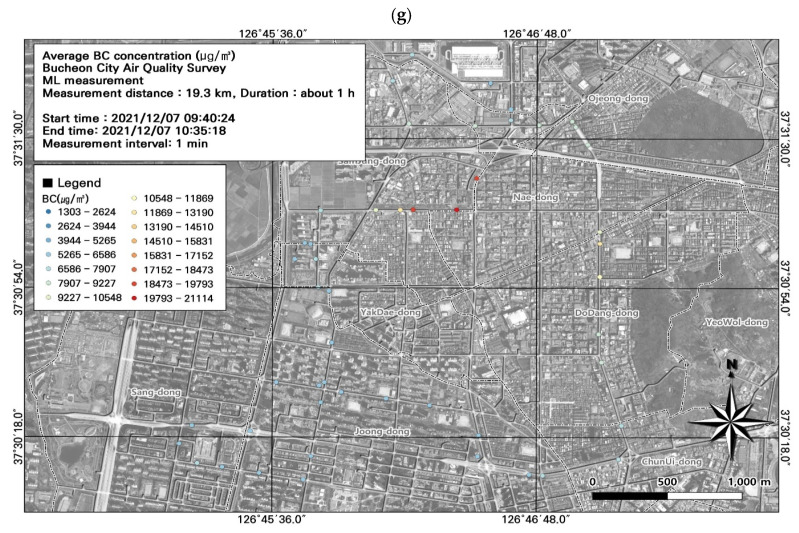
Spatial and temporal plots of ultrafine black carbon (BC) concentrations measured using the mobile laboratory (ML): (**a**) 6 December, first run; (**b**) 6 December, second run; (**c**) 6 December, third run; (**d**) 6 December, fourth run; (**e**) 6 December, fifth run; (**f**) 7 December, first run; (**g**) 7 December, second run.

Among the seven ML datasets, the highest average BC concentration was measured during the morning rush hour (8722.0 ± 2660.7 µg/m^3^) (Figure 9; Table 4), followed by the evening rush hour (7889.2 ± 3283.4 µg/m^3^). During commuting hours, the highest measurement was 16,139 µg/m^3^ at the Nae-dong intersection on December 6; on December 7, the highest daily BC concentration was 14,716 µg/m^3^. Overall, BC concentrations were higher during commuting hours. BC is a particulate pollutant used as an indicator of automobile emissions. However, BC concentrations are measured over 1 min intervals, and only average levels for the entire interval can be measured. Accordingly, it is necessary to consider the spatiotemporal distribution of BC in combination with PM, which can be measured at shorter intervals.

**Figure 10:**
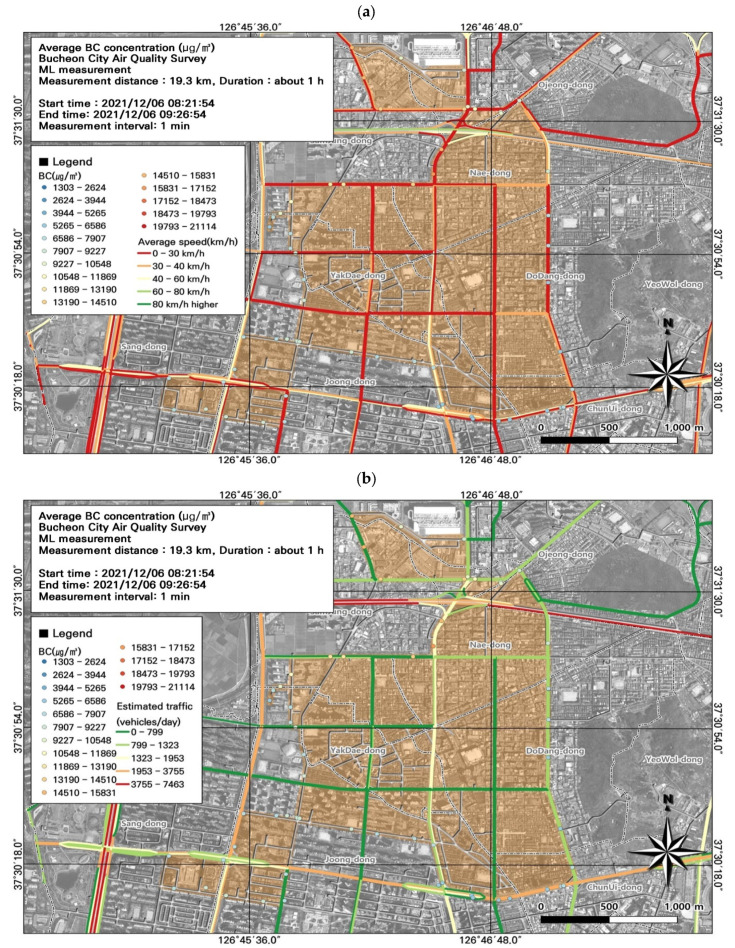
Association between black carbon (BC) concentrations and (**a**) average traffic speed and (**b**) estimated traffic volume during the morning rush hour from Figure 9a.

We subsequently performed a spatial analysis using the data during morning rush hour (Figure 9) to identify areas with particularly high BC concentrations (Figure 10). Because BC concentrations were measured at 1 min intervals, we could not perform a detailed comparison. Traffic was congested along the entire study route, and the average traffic speed was 0–30 km/h. Based on the comparison of traffic volume estimates and BC concentrations, the areas near Nae-dong and Gyeongin-ro had relatively high BC concentrations, and experienced elevated traffic volumes of 1323–1953 vehicles/day. Moreover, BC concentrations were relatively high at intersections. These findings highlight the importance of examining BC along with PM_10_ and PM_2.5_ when investigating vehicle emissions.

### 3.4. Spatiotemporal Patterns of NO_x_ Concentrations

**Figure 11:**
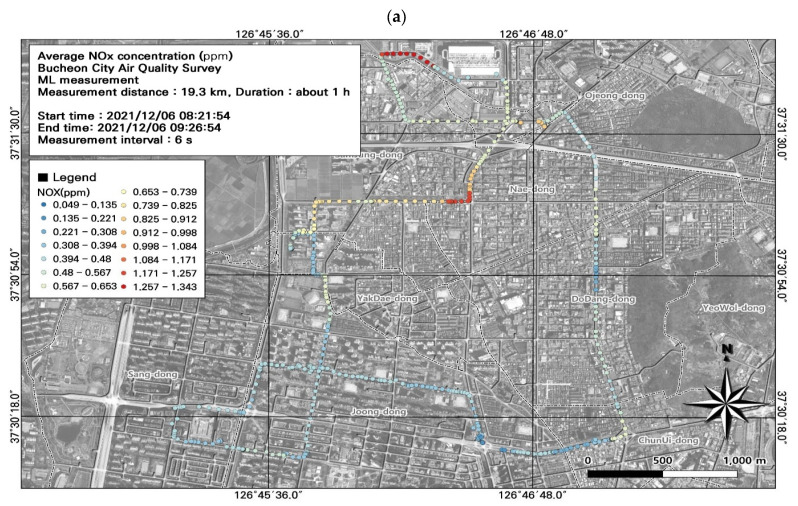

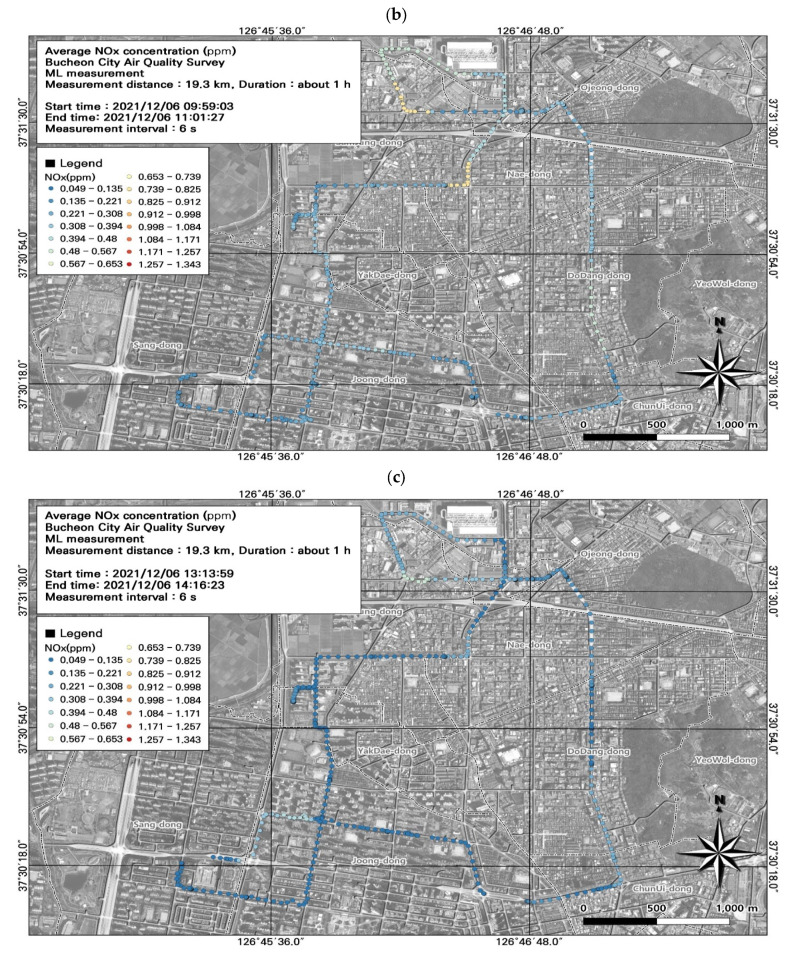

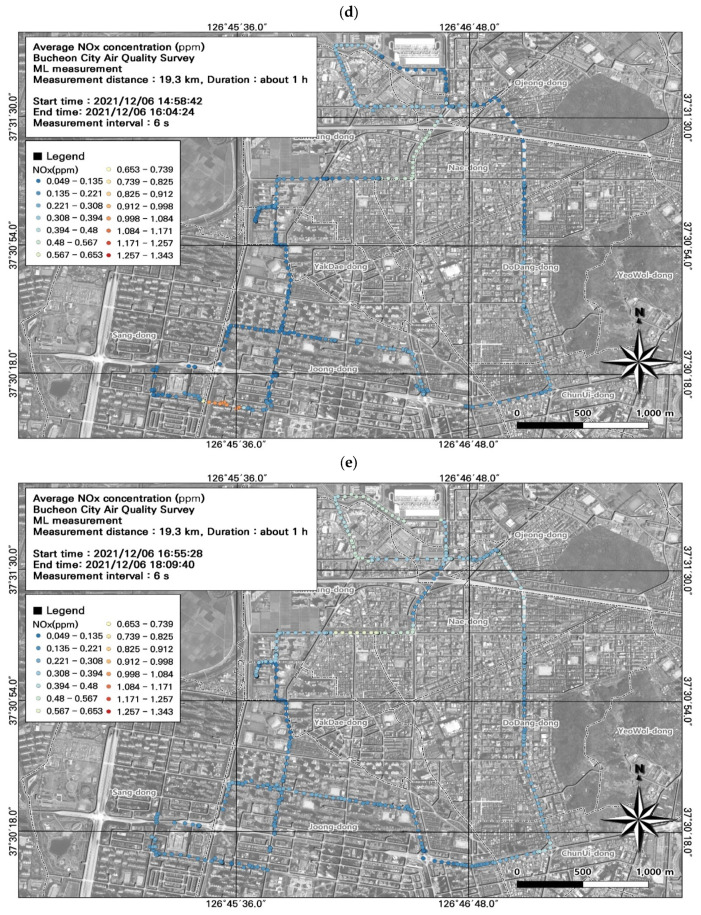

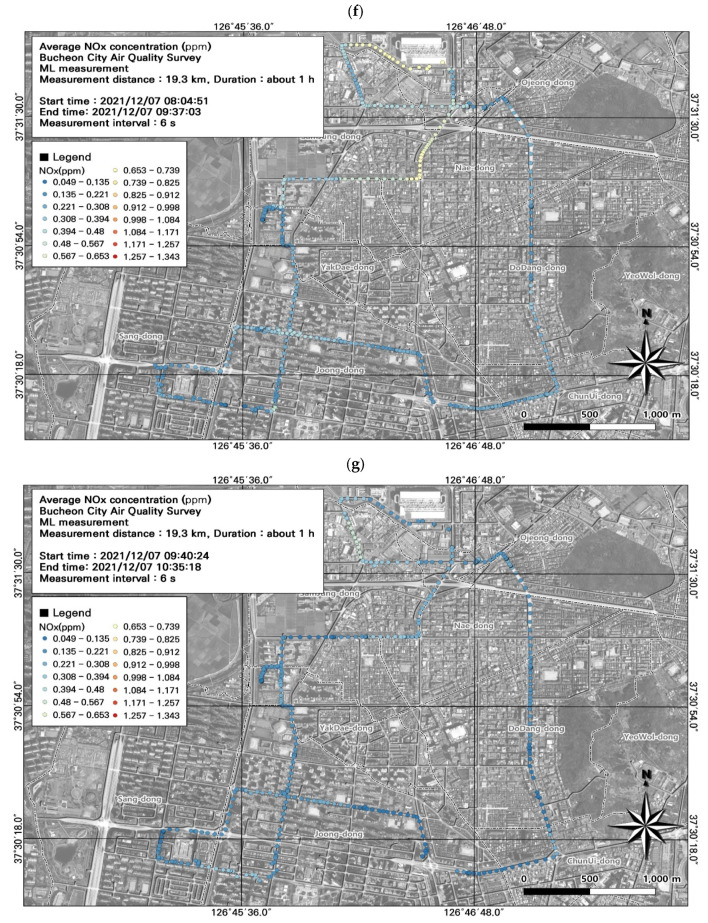
Spatial and temporal plots of nitrogen oxide (NO_x_) concentrations measured using the mobile laboratory (ML): (**a**) 6 December, first run; (**b**) 6 December, second run; (**c**) 6 December, third run; (**d**) 6 December, fourth run; (**e**) 6 December, fifth run; (**f**) 7 December, first run; (**g**) 7 December, second run.

NO_x_ concentrations are widely used as an indicator of road emissions. Our findings revealed that the 24 h average NO_2_ concentration (0.06 ppm) exceeded air quality standards. Among the seven ML datasets, the NO_x_ concentration was highest during the morning rush hour (0.50 ± 0.20 ppm), and the NO_2_ concentration was highest during the morning and evening rush hour (0.08 ± 0.03 ppm) (Figure 11; Table 5). The spatiotemporal distributions revealed high NO_x_ concentrations during commuting hours, with maximum concentrations of 1.34 ppm for NO_x_, 0.18 ppm for NO_2_, and 1.18 ppm for NO. Although the NO_x_ concentrations were measured at 1 s intervals, enabling a detailed spatiotemporal analysis, some points overlapped spatially; therefore, we modified the data to plot the NO_x_ values at 6 s intervals, the same as the PM_2.5_ and PM_10_ results.

**Figure 12:**
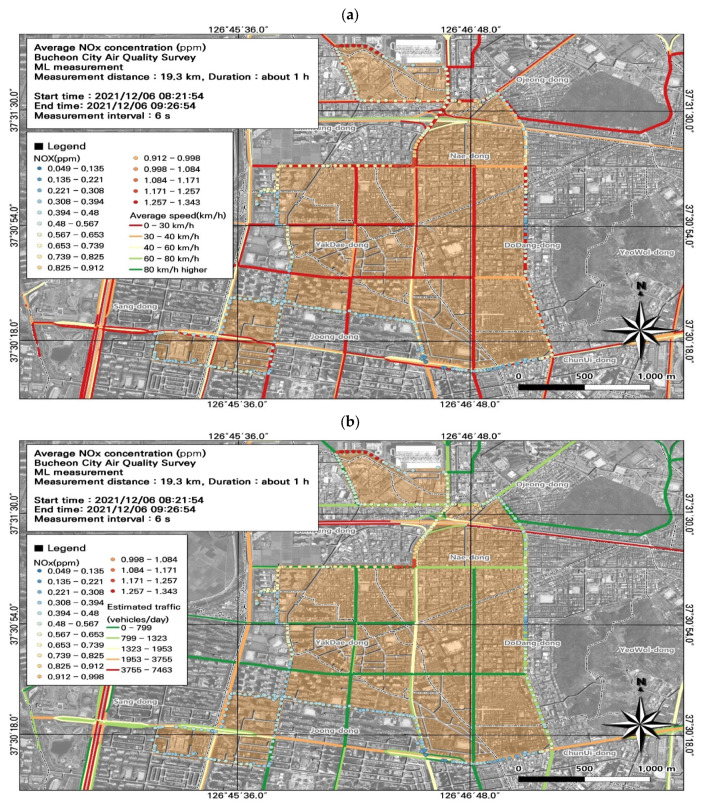
Association between nitrogen oxide (NO_x_) concentrations and (**a**) average traffic speed and (**b**) estimated traffic volume during the morning rush hour from Figure 11a.

As automobile exhaust is the main source of NO_x_ emissions, we examined the effects of traffic volume and average traffic speed on NO_x_ concentrations. Using morning rush hour data (Figure 11), we analyzed the spatial patterns of NO_x_ concentrations to identify hotspots (Figure 12). We identified hotspots of NO_x_ levels at road sections that experienced congestion during commuting hours, with average traffic speeds of 0–30 km/h. During commuting hours, the maximum NO_x_ concentration at the intersection near Nae-dong and Gyeongin-ro during rush hour was very high (1.34 ppm). Comparison of the estimated traffic volume and NO_x_ concentrations confirmed that the area experiencing high NO_x_ concentrations (i.e., the intersection near Nae-dong and Gyeongin-ro) also had an elevated traffic volume of 1323–1953 vehicles/day. These findings confirm the impact of vehicle emissions on NO_x_ emissions, and highlight the importance of pollution mitigation measures that consider traffic volume and average traffic speed.

### 3.5. GIS-Based Pollution Hotspot Analysis

In this study, each pollutant was classified, based on the average concentration over seven time periods, and the concentration at each point according to the movement of ML was quantitatively derived and visualized based on a GIS map. Based on this, spatial hotspots for each time zone can be derived. Our findings highlight the need for continuous management of targeted road sections with high pollutant concentrations. To realize such targeted measures, pollution hotspots must be identified based on spatiotemporal analyses of traffic-related variables for each pollutant (PM_10_, PM_2.5_, BC, NO_x,_ etc.) to inform policies. Future studies should comprehensively review target pollutants along with traffic speed (overall average and during congested periods) and volume, road levels, and other related variables to visualize air quality management measures based on GIS. Many local governments are already collecting real-time air quality data, but have not developed a system to visualize the data. Therefore, in order to effectively support air quality management measures, it is necessary to manage hotspots in the region using a GIS-based data visualization method.

## 4. Discussion and Conclusions

Public awareness of environmental issues, such as atmospheric fine dust pollution, is increasing. In Korea, AQMS and RAQMS networks have been installed in urban areas and along roads, respectively, to collect data on air pollutants, such as SO_2_, CO, NO_2_, O_3_, PM_10_, and PM_2.5_. However, the existing fixed RAQMS network is limited in the number of measurement sites available to record data. Accordingly, we used an ML to take measurements along roads in Bucheon, which has the highest population density in Gyeonggi-do, Korea. We analyzed the data and compared the results with local AQMS and RAQMS datasets. Unlike previous studies, we additionally used GIS data to compare the spatiotemporal distributions of individual pollutants against environmental variables.

This study was conducted in winter (December), which experiences among the highest seasonal fine dust concentrations. Because Bucheon does not have large-scale facilities with high emissions, atmospheric pollutants derived from fossil fuels (e.g., BC, NO, NO_2_, and NO_x_) are mainly emitted from mobile sources. We found that the ML-measured PM_10_ and PM_2.5_ concentrations were similar to those from the AQMS. Moreover, our spatiotemporal analysis indicated that vehicles were the main source of PM_10_ and PM_2.5_. Temporally, particulate and gaseous pollutant concentrations were high during commuting hours. Spatially, within the study area, the specially managed road had high pollutant concentrations. Our GIS-based visualization of pollution levels overlayed with road level, traffic (average and during congestion), and estimated traffic volume data simplified the identification of pollution hotspots. GIS enables the comparison of multiple environmental variables, and can support the establishment of air quality management measures. Although the instruments used in this study have first-grade measurement certifications, due to the limitations of light-scattering-based measurements, which can vary depending on the physicochemical characteristics of particles, we used the data for comparisons of regional spatiotemporal distribution to confirm the accuracy of measured concentrations.

During commuting hours, the maximum PM10 concentration reached 200.7 µg/m^3^ in the Nae-dong, Gyeongin-ro, and Ojeong-dong ready-mix concrete complex areas, and the maximum PM2.5 concentration was 161.7 µg/m^3^. The maximum NOx, NO2, and NO levels of 1.34 ppm, 0.18 ppm, and 1.18 ppm, respectively, were also detected during commuting hours. These findings support the need for targeted management of air pollution in this region, and highlight the benefit of comprehensively comparing road levels, driving speed, and traffic levels when identifying hotspots of air pollution. Such analyses will contribute to the development of air quality management measures customized to regional characteristics.

In this study, we used an ML to measure real-time air pollutant concentrations to identify high-concentration areas. Many local governments have begun to measure road pollution using MLs. However, to assist accurate decision making, it is necessary to take into account regional characteristics by visualizing, overlaying, and mapping pollution data with environmental and traffic variables to conduct comprehensive GIS-based analyses. We believe the results of this study will contribute to the air quality management policies of local governments in Korea, enabling the data-driven development of air quality management measures that target high-pollution areas. In the future, we plan to expand on the research in this study by investigating additional pollutants in additional regions.

## Figures and Tables

**Table 1:** Measurement items and devices.

Measurement Item	Instrument	Measurement Range	Interval
Latitude, Longitude, Altitude, Speed	GPS system	-	1 s
PM_10_, PM_2.5_	Grimm 11-D (Grimm Aerosol Technik)	0.253–35.15 μm	6 s
PM_2.5_	PMM-304 (APM)	0–200 µg/m^3^	60 s (1 m)
NO_x_	Serinus 40 (Ecotech)	0–20 ppm	1 s
Black carbon	AE51 aethalometer (Magee Scientific)	0–1000 µg/m^3^	60 s (1 m)

**Table 2:** Comparison of air pollutant measurements from the mobile laboratory (ML), Nae-dong air quality monitoring system (AQMS) station, and Songnae-daero roadside air quality monitoring system (RAQMS) station.

Measurement Item	Unit	ML	AQMS	RAQMS
PM_10_	µg/m^3^	78.7 ± 26.2	85.1 ± 21.5	63.5 ± 12.6
PM_2.5_	µg/m^3^	55.7 ± 13.0	52.7 ± 15.1	38.9 ± 8.3
Black carbon	µg/m^3^	6205.7 ± 3020.1	-	-
NO	ppm	0.21 ± 0.16	-	-
NO_2_	ppm	0.07 ± 0.03	0.06 ± 0.00	0.05 ± 0.01
NO_x_	ppm	0.28 ± 0.18	-	-
Temperature	°C	-	6.4 ± 3.8	-
Relative humidity	%	-	60. 6 ± 13.7	-
Wind speed	Km/h	-	0.4 ± 0.3	-

**Table 3:** PM10 and PM2.5 average concentration in the mobile laboratory (ML) during seven measurement runs.

Number of Measurements	PM_10_ (µg/m^3^)	PM_2.5_ (µg/m^3^)
1	79.6 ± 19.7	62.7 ± 13.0
2	69.1 ± 21.6	52.6 ± 11.7
3	57.1 ± 38.8	39.5 ± 14.2
4	63.6 ± 34.5	42.9 ± 11.5
5	91.8 ± 17.6	59.2 ± 8.8
6	71.2 ± 13.8	59.5 ± 6.8
7	67.4 ± 25.8	57.6 ± 17.4

**Table 4:** BC average concentration in the mobile laboratory (ML) during seven measurement runs.

Number of Measurements	BC (µg/m^3^)
1	8722.0 ± 2660.7
2	6005.6 ± 2197.0
3	3300.2 ± 1780.7
4	4580.8 ± 3064.2
5	5624.0 ± 2016.2
6	7187.2 ± 2321.0
7	7889.2 ± 3283.4

**Table 5:** NOx average concentration in the mobile laboratory (ML) during seven measurement runs.

Number of Measurements	NO (ppm)	NO_2_	NOx (ppm)
1	0.43 ± 0.18	0.08 ± 0.03	0.50 ± 0.20
2	0.24 ± 0.12	0.07 ± 0.03	0.31 ± 0.14
3	0.09 ± 0.09	0.06 ± 0.02	0.15 ± 0.10
4	0.14 ± 0.13	0.07 ± 0.05	0.21 ± 0.17
5	0.20 ± 0.11	0.08 ± 0.03	0.28 ± 0.12
6	0.21 ± 0.12	0.06 ± 0.02	0.27 ± 0.14
7	0.15 ± 0.08	0.05 ± 0.02	0.20 ± 0.08

## Data Availability

The original data presented in the study are included in the article; further inquiries can be directed to the corresponding author.
